# Antifungal Activity of Denture Soft Lining Material Modified by Silver Nanoparticles—A Pilot Study

**DOI:** 10.3390/ijms12074735

**Published:** 2011-07-22

**Authors:** Grzegorz Chladek, Anna Mertas, Izabela Barszczewska-Rybarek, Teresa Nalewajek, Jarosław Żmudzki, Wojciech Król, Jan Łukaszczyk

**Affiliations:** 1 Department of Materials Technology, Silesian University of Technology, ul. Krasińskiego 8, 40-019 Katowice, Poland; E-Mail: Jaroslaw.Zmudzki@polsl.pl; 2 Chair and Department of Microbiology and Immunology in Zabrze, Medical University of Silesia in Katowice, ul. Jordana 19, 41-808 Zabrze, Poland; E-Mails: amertas@sum.edu.pl (A.M.); teresa-nalewajek@wp.pl (T.N.); wkrol@sum.edu.pl (W.K.); 3 Department of Physical Chemistry and Technology of Polymers, Silesian University of Technology, M. Strzody 9, 44-100 Gliwice, Poland; E-Mails: Izabela.Barszczewska-Rybarek@polsl.pl (I.B-R.); jan.lukaszczyk@polsl.pl (J.Ł.)

**Keywords:** nanotechnology in dentistry, silver nanoparticles, antifungal, dental materials, soft liner, *Candida albicans*

## Abstract

Soft liner materials in oral cavity environments are easily colonized both by fungi and dental plaque. These factors are the cause of mucosal infections. The microorganism that most frequently colonizes soft liner materials is *Candida albicans*. Colonization occurs on the surface of materials and within materials. A solution to this problem might involve modification of soft liner materials with silver nanoparticles (AgNPs). In this article, we present results showing the antifungal efficacy of silicone soft lining materials modified with AgNPs. The modification process was conducted by dissolving both material components (base and catalyst) in a colloidal solution of AgNPs and evaporating the solvent. Composites with various AgNP concentrations (10, 20, 40, 80, 120 and 200 ppm) were examined. The *in vitro* antifungal efficacy (AFE) of composite samples was 16.3% to 52.5%.

## 1. Introduction

Denture wearing favors the occurrence of stomatopathy by increasing both the number of local injuries and the time that the mucosa is in contact with microorganisms [1,2]. Budtz-Jorgensen *et al.* [3] report that 50% of complete and partial denture wearers experience problems with stomatopathy. In contrast, 93.8% of patients with stomatopathy also harbor isolated fungi [4]. Soft liner materials are usually used for denture relining to evenly distribute the loads transferred onto soft tissues during motion. Soft structures should increase the comfort of denture wearers and support prosthetic treatment. They are primarily used in patients with thin atrophic mucosa, in patients with normal mucosa with an atrophied ridge, in patients with a sharp alveolar ridge and when the mucosa exhibits a low tolerance to the load applied by the dentures [5,6]. The specific mechanical characteristics of soft liners facilitate their use in other non-conventional applications [7].

*In vitro* studies covering longer time periods show that the use of soft liners might intensify the formation of fungal biofilms [8,9]. Colonisation of soft liners by *Candida albicans* is favored by the presence of saliva and serum pellicles [10–13]. Fungal adhesion to material surfaces is the first step of colonisation [14,15]. Fungi can then penetrate the inside of the material [8,16]. This phenomenon is particularly unfavorable because it significantly reduces the possibility of efficient denture disinfection with surface-active agents available to patients; however, numerous reports have confirmed the biocidal effectiveness of silver nanoparticles (AgNPs) in dental applications [17–23]. Moreover, silver activity rarely causes resistant microorganisms to develop [24]. It can be hypothesised that the modification of soft liner materials with AgNPs has the potential to decrease the risks associated with the colonisation of soft liners by pathogenic fungi.

In this study, we present a method of incorporating AgNPs into the chemically cured silicone soft liner material Ufi Gel SC (UG). The aim of this work was also to evaluate the antifungal efficacy of these developed composites.

## 2. Results and Discussion

### 2.1. Material Preparation

**Figure 1:**
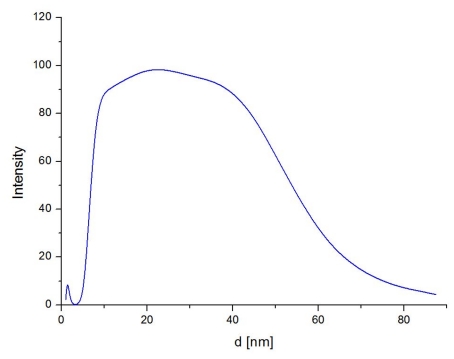
Silver nanoparticle (AgNP) size distribution in 30 ppm solution.

Measurements using dynamic light scattering (DLS) for colloidal solutions of 30 ppm AgNPs showed an average NP size of 22.8 nm. The size distribution of AgNPs is presented in Figure 1.

No hexane from the modification process was detected on ^1^H NMR spectra made for samples of composites, base UG/AgNPs and catalyst UG/AgNPs.

**Figure 2:**
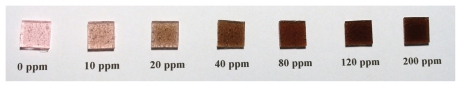
Polymerized material samples: Ufi Gel SC (UG) and composites with different AgNP concentrations.

Figure 2 shows polymerised samples of commercially used materials with different AgNP concentrations. It is clear that when the AgNP concentration increased, the color of the samples became darker.

**Figure 3:**
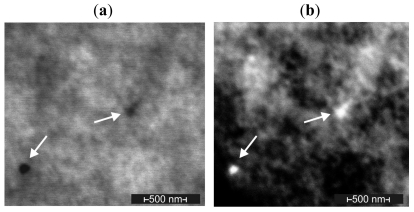
Micrographs showing AgNPs in a composite with 160 ppm AgNPs. Scanning transmission electron image using (**a**) the wet-STEM detector and (**b**) the gaseous secondary electron detector (GSED).

Scanning electron microscopy measurements show the presence of both individual particles (Figure 3) and large nanoparticle agglomerates in the modified soft liners. Individual particles from all specimens usually ranged from 10 to 30 nm. In the case of a higher AgNP concentration, a greater number of nanoparticle aggregations and larger sized aggregations were observed. Starting with a concentration of 80 ppm, numerous large agglomerates were noted. Most of them ranged between 100 and 300 nm, but there were also huge aggregations that exceeded 1 μm, and individual AgNPs were still observed.

### 2.2. Antifungal Activity of Studied Materials

**Figure 4:**
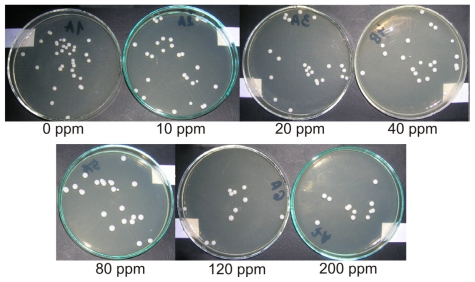
Example results of antifungal tests against *Candida albicans* after 17 h of incubation with UG and UG SC/AgNP composites.

Antifungal test results are presented in Table 1 and Figure 4. Positive and negative controls yielded expected results. For material specimens without AgNPs, the observed *Candida albicans* CFU/mL value increased by 23.4% compared with the positive control. The introduction of 10 ppm AgNPs to the polymer led to an antifungal efficacy (AFE) of 16.4%. Increasing the AgNP concentration in the composite to 20 ppm resulted in an additional increase in the AFE of 8%. The average AFE value for samples with 40 ppm AgNPs was 31.5%. An additional increase in the AgNP concentration resulted in a less dynamic, but still visible, AFE increase. For the highest examined AgNP concentration (200 ppm), the maximum composite AFE value reached was 52.2%.

### 2.3. Discussion

A soft liner material with antifungal characteristics has not yet been developed. Although Nikawa *et al.* [10,25] reported an inhibitory effect on *Candida albicans* for Molloplas B, other authors [5,26] argue that well-polymerised material does not have these types of properties. Pavan *et al.* [26] reported that both Ufi Gel P and Malloplast B do not show any inhibition, although adhesion of *Candida albicans* is higher in the case of Ufi Gel P. Bulad *et al.* [5] examined six types of soft liner materials (including hard Uif Gel C) and analysed colonisation and penetration by *Candida albicans*. None of the examined materials showed inhibition of *Candida albicans* compared with a positive control. There was also no observed difference in adhesion of *Candida albicans* between these various materials. Ufi Gel C was proved to be less resistant to penetration by *Candida* blastospores than the other tested materials, which was explained by the effect of material porosity. The absence of hyphal forms of *Candida albicans* inside the materials was associated with higher hardness; all remaining materials that were classified as soft were also penetrated to a significant extent by hyphal formations of *Candida albicans*. Moreover, other studies [5,14,27] showed that specimens with smooth surfaces are colonized much more slowly by fungi, although some other studies do not confirm such a trend [25,26]. Soft liner porosity increases with denture wearing time, which might increase the susceptibility of those types of materials to colonisation by microorganisms [28]. In contrast, the efficacy of AgNPs against *Candida albicans* was confirmed by numerous studies [29–32]. Nevertheless, there are no studies to date attempting to modify soft liners with silver AgNPs.

The present modification method for silicone soft liners makes it possible to incorporate AgNPs into materials and completely evaporate hexane from base and catalyst solutions. Microscopy investigations showed individual particles and large nanoparticle agglomerates inside the obtained materials. For the 80 ppm AgNP concentration, numerous large and very large aggregations were observed. Kvitek *et al.* [33] reported that aggregation of the AgNPs reduce effective surface of AgNPs which can contact with microorganisms and decrease the antimicrobial effect.

In an environment containing specimens made of UG (without AgNP content) there was an observed increase of 23.4% in the CFU/mL value of *Candida albicans* compared with a positive control. These results correspond with the results of other authors, who did not show any antifungal activity of this material [5,26]. Observed AFE values in relation to *Candida albicans* for the modified material were from 16.3% to 52.2%. Attention should be paid to the high value of the standard deviation (SD) for the 10 ppm concentration. In cases of AgNP concentrations above 20 ppm, the repeatability of the results was remarkably better. Increasing the AgNP content in the composites resulted in an increased AFE; that increase was especially visible in the case of the 40 ppm concentration. A five-fold increase in the AgNP concentration, from 40 to 200 ppm, increased the AFE by a further 28%. A similar tendency was previously shown [23] using the authors’ own method of AgNP introduction into chemical-cure acrylic dental resin by means of AgNP in situ synthesis using silver benzoate (AgBz). The resins were tested *in vitro* for antibacterial activity against *Streptococcus mutans*. Resin containing 0.2% (w/w) AgBz showed 52.4% bacterial inhibition. A 1.5-fold increase in the AgBz concentration caused an increase in the bacterial inhibition by a further 45.1%. A lack of linear dependence between the quantity of the introduced AgNPs and antimicrobial efficacy of the examined material denote, in practice, that there is a need to define an optimum level above which any increase in the AgNP concentration will be inexpedient. For that reason, the results of fungicidal tests should be correlated with other effects of material modification. For instance, the analysed materials experienced a color change (an important functional property of dental materials) resulting from the plasmon effect of the AgNPs [23]. This phenomenon was very significant for AgNP concentrations above 80 ppm. Additionally, studies reported that AgNPs are cytotoxic to different cell lines [34]. This toxicity is only partially recognized [35]. Results [36] showed that AgNPs was cytotoxicity in the case of exposure at high concentrations. Kvitek *et al.* [33] reported that the AgNPs having the diameter of 25 nm caused death of the human fibroblasts at the concentrations higher than 60 mg/L. The authors concluded that AgNPs do not generate any danger in applications, but only if the concentration is retained (it should be sufficient for the inhibition of microorganism growth). These results suggest that tested composites with concentrations to 40 ppm of AgNPs should be safe in an oral cavity environment, but this assumption should be clearly confirmed in further investigations. Fan *et al.* [23] also reported the ability to change the mechanical characteristics of the modified materials by increasing the AgNP concentration. Moreover, composites with a high AFE could have a negative influence on the physiological microflora in the oral cavity. However, low AFE values obtained in the environment surrounding specimens with concentrations from 20 to 40 ppm should eliminate fungi on the surface and inside of the material. The aforementioned assumption requires confirmation with experiments conducted under *in vivo* conditions.

## 3. Experimental Section

### 3.1. Materials Preparation

Chemically cured silicone soft liner (Ufi Gel SC) material was used for all studies (VOCO GmbH, Cuxhaven, Germany).

AgNP colloids (concentration of 1000 ppm) in hexane (AMEPOX Co. Ltd, ŁódŸ, Poland) were diluted in 95% n-hexane (POCH S.A., Gliwice, Poland) down to a concentration of 30 ppm. The AgNP sizes in the colloidal solutions were then determined using dynamic light scattering (DLS) spectroscopy (Brookhaven). The particle hydrodynamic dimensions in solution were assessed by performing measurements at a single angle of θ = 90°.

The UG base and the UG catalyst were next dissolved in hexane, achieving a concentration of 7% (w/w). Dissolution was performed in 300 mL Erlenmeyer flasks by stirring with a magnetic stirrer at room temperature for 2 h.

The AgNP suspension mass in hexane necessary for a composite with a particular concentration was calculated according to the following equation:

(1) $$m_{Ag-hex}=\frac{c_{Ag}\times m_{UG}\times 10^{6}}{c_{Ag-heks}(10^{6}-c_{Ag})}$$

where mAg-hex was the AgNP suspension mass [g], cAg was the AgNP concentration in a composite component UG/AgNP [ppm], mUG was the UG component mass [g] and cAg-hex was the Ag concentration in the hexane suspension (30 ppm).

Before weighing, the AgNP suspensions were stirred with a magnetic stirrer for 15 min. Then, the amount of suspension was calculated according to equation (1) and added to the solution of modified material components. The obtained mixture was stirred with a magnetic stirrer for 15 min.

To remove hexane from the solution, a two-step procedure was used. The hexane was preliminarily evaporated from the flask by placing it in a rotary evaporator (IKA RV-10) for 15 min. The pressure was reduced to 100 mbar. Then, the preliminarily condensed suspension was poured into a Petri dish and warmed in a dryer at 50 °C for 24 h. Moving the modified material to dishes was advantageous because of the additional operations related to specimen polymerisation; the working time of the material is ~2 min, whereas taking modified components out of the flask after complete solvent evaporation requires a great deal of time and is usually associated with significant material losses. In order to evaluate the effectiveness of the evaporation procedure, the ^1^H NMR spectra were used (NMR spectrometer, UNITY INOVA, Varian, 300 MHz).

Following the previously described procedure, both the base and the catalyst of the material were modified. Samples were prepared with the following AgNP concentrations: 10, 20, 40, 80, 120 and 200 ppm.

The modified material samples were mixed together in a mass ratio of 1:1 and polymerised according to the manufacturer’s procedure. During polymerisation, composites were placed between two glasses separated by 2.3 mm thick dividers. From the plates of polymerised material, square specimens that measured 10 mm × 10 mm and were 2.3 mm thick were cut. Before microbiological testing, the specimens were plasma sterilised.

SEM measurements were performed on a Quanta 250 ESEM FEG scanning electron microscope (FEI Company) operating at 30 kV in environmental mode, using the wet-STEM detector to detect STEM images in SEM and the gaseous secondary electron detector (GSED). The chamber pressure was 10 mmHg.

A solid triangular sample of silicon was frozen in liquid nitrogen and mounted directly onto a specimen holder in the cryo-chamber of an ultramicrotome (Leica, EM UC7) which was then cooled down to −120 °C. Trimming with a glass knife helped to prepare the plane from which the sample was cut.

Sections were cut at −120 °C with a speed of 5 mm/sec and the section thickness set to 200 nm. The thin leafs of silicon were manipulated on the Cu grid using an eyelash probe, and the sample was squeezed between ceramic surfaces and directly transferred using the high-vacuum technique to a glass holder adapted for drying. The samples were stored under vacuum until imaging.

### 3.2. Antifungal Test

The *in vitro* antifungal activities of UG modified with silver nanoparticles were examined according to Xu *et al.* [37]. A standard strain of *Candida albicans,* ATCC 10231, was used. Samples of UG and UG/AgNPs with nanosilver particles in concentrations from 10 to 200 ppm were tested. Samples with and without AgNPs were introduced into 4 mL of fungal suspension in triptonic water, containing approximately 1.5 × 10^5^ colony forming units (CFUs) of *Candida albicans* in 1 mL. A suspension of 1.5 × 10^5^ CFU/ml of *Candida albicans* in triptonic water was tested as a positive control (blank). Samples of UG (without AgNPs) in triptonic water without *Candida albicans* and pure triptonic water were tested as negative controls. All mixtures were incubated at 37 °C in static conditions for 17 h. After incubation, 20 μL of each mixture was seeded onto a Sabouraud agar plate. These plates were incubated at 37 °C for an additional 48 h. Then, the number of *Candida albicans* colonies was counted, and the material AFE was calculated according to the following equation:

(2) $$AFE\;[\%]=\frac{V_{c}-V_{t}}{V_{c}}\times 100\%$$

where V_c_ was the number of viable fungal colonies of the positive control (BLANK) and V_t_ was the number of viable fungal colonies of the test specimen.

## 4. Conclusions

In this study, we presented a method of AgNP incorporation into chemically cured silicone soft liner materials. The AFE of the achieved composites containing 10 to 200 ppm AgPNs was 16.3% to 52.5%. This level of AFE should be capable of preventing colonisation of *Candida albicans* on soft denture linings. Future research should examine the mechanical characteristics of the achieved composites and confirm their microbiological characteristics in *in vivo* studies.

## Figures and Tables

**Table 1:** The average antifungal effect for UG and UG/AgNP composites against *Candida albicans*. CFU, colony forming units; AFE, antifungal efficacy; V_t_, the number of viable fungal colonies of the test specimen; SD, standard deviation.

AgNPs ppm	CFU/mL (V_t_) ×10^3^	SD ×10^3^	AFE	SD
**0**	1.43	0.22	0%	-
**10**	0.96	0.16	16.3%	6.2%
**20**	0.87	0.04	24.2%	3.5%
**40**	0.79	0.04	31.5%	3.1%
**80**	0.74	0.06	36.6%	5.4%
**120**	0.60	0.07	47.8%	6.3%
**200**	0.55	0.07	52.2%	6.2%
**Vc** (**BLANK)**	**1.15**	-	-	-
